# Chloroplast Genome Characterization, Comparative Analysis, and Phylogenetic Insights into Five *Aegilops* Species

**DOI:** 10.3390/ijms27135680

**Published:** 2026-06-24

**Authors:** Shyryn Almerekova, Moldir Yermagambetova, Sayagul Turemuratova, Shynar Anuarbek, Minura Yessimbekova, Shun Sakuma, Yerlan Turuspekov

**Affiliations:** 1 Institute of Plant Biology and Biotechnology, Almaty 050040, Kazakhstan; almerekovakz@gmail.com (S.A.); ermaganbetova.moldir@bk.ru (M.Y.); saya_kh@mail.ru (S.T.); shinar_anuar92@mail.ru (S.A.); 2 Faculty of Biology and Biotechnology, Al-Farabi Kazakh National University, Almaty 050040, Kazakhstan; 3 Kazakh Scientific Research Institute of Agriculture and Plant Growing, Almaty 040909, Kazakhstan; minura.esimbekova@mail.ru; 4 Faculty of Agriculture, Tottori University, Tottori 680-8550, Japan; ssakuma@tottori-u.ac.jp

**Keywords:** goatgrasses, plastome, nucleotide diversity, simple sequence repeats, phylogenomics, maternal lineage

## Abstract

The genus *Aegilops* comprises important wild relatives of cultivated wheat and represents a valuable genetic resource for wheat improvement. In this study, the complete chloroplast genomes of five *Aegilops* species (*Ae. crassa*, *Ae. cylindrica*, *Ae. juvenalis*, *Ae. tauschii*, and *Ae. triuncialis*) collected from Kazakhstan and Uzbekistan were sequenced, assembled, and comparatively analyzed. The chloroplast genomes exhibited a conserved quadripartite structure consisting of a large single-copy (LSC), a small single-copy (SSC), and two inverted repeat (IR) regions. Genome sizes ranged from 135,612 to 136,840 bp, with an identical GC content of 38% across all species. Comparative analyses revealed high structural conservation among chloroplast genomes, particularly within IR regions, whereas greater sequence divergence was observed in the non-coding regions of the LSC and SSC. Sliding-window analysis identified several highly polymorphic regions, including *rpl32-trnL(UAG)*, *ndhF-rpl32*, *trnC(GCA)-rpoA*, *psbA*, and *ndhD*, which may serve as potential DNA barcodes and informative markers for phylogenetic studies. A total of 850 chloroplast simple sequence repeats (SSRs) were detected, predominantly A/T-rich mononucleotide repeats. Codon usage analysis demonstrated a conserved preference for A/U-ending codons across all species. Ka/Ks analysis indicated that most chloroplast protein-coding genes are under strong purifying selection, although relatively elevated evolutionary rates were detected in *rpoA* and *ycf4*. Phylogenetic analyses based on complete chloroplast genomes strongly supported sectional relationships within *Aegilops* and confirmed close maternal relationships among several species. Overall, this study provides chloroplast genome resources for *Aegilops* and contributes to understanding chloroplast genome evolution, phylogeny, and molecular marker development.

## 1. Introduction

The genus *Aegilops* L. (goatgrass), a member of the family Poaceae Barnhart, comprises 23 currently accepted species [1]. These species are distributed predominantly across the Mediterranean basin, Western and Central Asia, and neighboring regions [2,3,4,5]. *Aegilops* is notable for its considerable genomic diversity, encompassing the U, C, M, N, S, and D genomes [6]. Taxonomically, the genus is divided into five sections: *Sitopsis* (Jaub. & Spach) Zhuk., *Vertebrata* Zhuk. emend. Kihara, *Cylindropyrum* (Jaub. & Spach) Zhuk., *Comopyrum* (Jaub. & Spach) Zhuk., and *Aegilops*. In Kazakhstan, five *Aegilops* species have been recorded: *Ae. cylindrica* Host, *Ae. crassa* Boiss., *Ae. juvenalis* (Thell.) Eig, *Ae. tauschii* Coss., and *Ae. triuncialis* L. [7]. These species belong to the sections *Vertebrata*, *Cylindropyrum*, and *Aegilops* [2,3]. These *Aegilops* species exhibit diverse ploidy levels ranging from diploid (*Ae. tauschii*, 2n = 2x = 14) to tetraploid (*Ae. cylindrica*, *Ae. triuncialis*, and *Ae. crassa*, 2n = 4x = 28) and hexaploid (*Ae. juvenalis*, 2n = 6x = 42) forms, with distinct combinations of nuclear and organellar genomes reflecting their complex evolutionary origins through interspecific hybridization [8,9,10]. Also, these species differ in morphological characteristics, including spike structure, the presence and length of awns, and spike density and pubescence. *Ae. cylindrica*, belonging to the section *Cylindropryum*, has a narrow cylindrical spike. The glumes are rough, with two teeth on the lower spikelets: one short and wide, the other in the form of a spear-like end or awn. The upper spikelet has two teeth and an awn up to 2.5–7 cm long. *Ae. triuncialis*, a member of the *Aegilops* section, has spike scales with 2–4 awns. The glumes are glabrous, rough along the veins, with 2–3 spines on the lower spikelets and 3 curved spines on the upper spikelets. *Ae. crassa*, *Ae. juvenalis*, and *Ae. tauschii*, which belong to the section *Vertebrata*, despite some morphological similarities, are characterized by significant differences. For example, *Ae. crassa* has hairy glumes with 2 teeth at the top: one is short and wide, the other is narrow and has an awn-like end. In *Ae. juvenalis*, the glumes are appressed-hairy, with 2–4 awns. *Ae. tauschii* has glumes without teeth or awns, blunt at the top, with a thickened edge, smooth or rough [5,11].

*Aegilops* species possess diverse adaptive traits, including tolerance to drought, salinity, and extreme temperatures, as well as resistance to various pathogens, making them valuable genetic resources for wheat improvement [12,13,14,15]. As wild relatives of cultivated wheat, *Aegilops* species have played an important role in the evolution and breeding of wheat [16]. Among them, *Ae. tauschii* is important because it has been identified as the donor of the D genome of common bread wheat, *Triticum aestivum* L. [17]. Owing to their close phylogenetic relationship with cultivated wheat, *Aegilops* germplasm is widely used in breeding and genetic studies, and its conservation and genetic characterization are essential for identifying useful alleles that may support the development of stress-resilient wheat cultivars.

Phylogenetic relationships within *Aegilops* have been investigated mainly in the context of its close evolutionary relationship with *Triticum*, particularly because several *Aegilops* species are considered important genome donors and wild relatives of wheat [18]. Chloroplast non-coding regions have been used to resolve intra- and interspecific relationships among diploid *Triticum-Aegilops* species [19], while the chloroplast *trnT-F* region has been applied to infer relationships among Triticum and *Aegilops* species as potential genome progenitors of bread wheat [20]. Bordbar with coauthors [21] examined D-genome-bearing *Aegilops* and *Triticum* species from Iran and adjacent regions using nuclear microsatellites, ITS, and chloroplast *trnL-F* sequences. The study revealed high genetic diversity in *Ae. tauschii*, two distinct gene pools within this species, and a close relationship among *Ae. crassa*, *Ae. juvenalis*, and *Ae. vavilovii*. DNA barcoding approaches have also been applied to *Aegilops*, using markers such as ITS, *matK*, *rbcL*, *trnH-psbA*, and p*sbM-petN* for species identification and phylogenetic analysis [22,23]. Overall, these studies demonstrate the usefulness of both plastid and nuclear markers for resolving phylogenetic relationships, assessing genetic diversity, and clarifying species boundaries in *Aegilops*. However, the resolution of a single or a limited number of barcode loci may be insufficient for closely related taxa, especially in groups with complex evolutionary histories involving hybridization and polyploidization [22].

Chloroplasts are essential plant organelles that contain their own genomes [24]. In most land plants, the chloroplast genome, or plastome, has a conserved quadripartite structure consisting of a large single-copy region (LSC), a small single-copy region (SSC), and two copies of inverted repeat regions (IRs) [25]. Advances in high-throughput sequencing technologies have enabled the rapid, large-scale sequencing of complete chloroplast genomes, facilitating their use as “super-barcodes” for species identification and taxonomic studies across many plant taxa [26,27,28]. Plastome nucleotide sequences are also highly informative for resolving phylogenetic relationships among plant groups [25,29] and for identifying highly variable regions that can be developed as DNA barcodes for particular genera or species complexes [30]. However, despite the increasing availability of plastome data, only a limited number of comparative chloroplast genome studies have been conducted in *Aegilops* species [31,32,33,34]. In a previous study, the *rpl32-trnL-UAG*, *ccsA-ndhD*, *rbcL-psaI*, and *rps18-rpl20* regions were identified as highly variable among 17 *Ae. tauschii* accessions [31]. Another study identified *trnK-UUU*, *trnK-UUU–rps16*, *rps16–trnQ*, *trnQ*, and *psbE–petL* as hypervariable regions across chloroplast genomes of the *Triticum-Aegilops* complex [34]. The chloroplast genome scale analyses of the *Triticum-Aegilops* complex have provided greater resolution of maternal phylogenetic relationships and revealed major plastid lineages associated with the A, B, and D nuclear genomes [33]. Thus, complete chloroplast genome data represent an important resource for identifying informative markers for species delimitation, improving phylogenetic resolution, and assessing genetic variation in *Aegilops* and related taxa.

Various molecular markers, including AFLP, ISSR, SSR, SNP, iPBS, and SCoT markers, have been used to investigate genetic diversity and population structure in *Aegilops* species [35,36,37,38,39,40,41,42,43,44,45,46,47]. In addition, chloroplast genomes are a valuable source of SSR markers for population genetic, phylogeographic, and conservation studies [48,49,50,51].

In the present study, we sequenced, assembled, and annotated the complete chloroplast genomes of five *Aegilops* species, namely *Ae. cylindrica*, *Ae. crassa*, *Ae. juvenalis*, *Ae. tauschii*, and *Ae. triuncialis*. These newly generated plastome sequences were used to perform comparative genomic analyses, identify highly polymorphic regions suitable as candidate DNA barcodes, detect simple sequence repeats (SSRs) as potential molecular markers for population genetic studies, and reconstruct phylogenetic relationships within the genus *Aegilops*. In addition, this study expands the available chloroplast genome resources for Central Asian *Aegilops* germplasm and provides a comparative assessment of plastome variation among representative species from this region.

## 2. Results

### 2.1. General Characteristics of Chloroplast Genomes Among Aegilops Species

**Figure 1:**
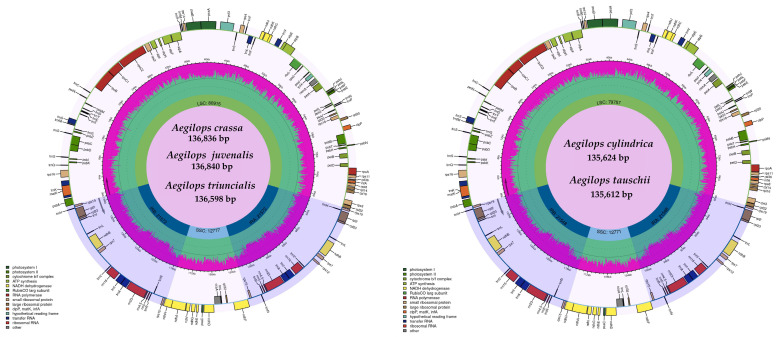
Chloroplast genome map of five *Aegilops* species (*Ae. crassa*, *Ae. cylindrica*, *Ae. juvenalis*, *Ae. tauschii*, and *Ae. triuncialis*) sequenced in this study. The inner circles represent genome features, where the green ring indicates GC content, and the colored sectors correspond to genomic regions: LSC (large single-copy), SSC (small single-copy), and IR (inverted repeats).

The chloroplast genomes of the five *Aegilops* species (*Ae. crassa*, *Ae. cylindrica*, *Ae. juvenalis*, *Ae. tauschii*, and *Ae. triuncialis*) exhibited the typical quadripartite structure, consisting of an LSC, an SSC, and a pair of IR (IRa and IRb) regions. Genome sizes were highly conserved, ranging from 135,612 bp in *Ae. tauschii* to 136,840 bp in *Ae. juvenalis* (Table 1; Figure 1; Figure S1). The LSC region ranged from 79,745 to 80,921 bp, the SSC region from 12,771 to 12,797 bp, and each IR region from 21,548 to 21,572 bp. All genomes showed an identical GC content of 38%.

A total of 127–128 genes were annotated in these chloroplast genomes, including 82–83 CDSs, 37 tRNAs, and 8 rRNAs. Specifically, *Ae. cylindrica* and *Ae. tauschii* contained 127 genes (82 CDS), whereas *Ae. crassa*, *Ae. juvenalis*, and *Ae. triuncialis* contained 128 genes (83 CDS). Among these, several genes were duplicated in the IR regions, including 6–7 CDS genes, 8 tRNA genes, and 4 rRNA genes (Table 1). The *rps3* gene exhibited duplication in three species (*Ae. crassa*, *Ae. juvenalis*, and *Ae. triuncialis*), while occurring as a single-copy gene in the other two species (*Ae. cylindrica* and *Ae. tauschii*).

The chloroplast genomes of the five *Aegilops* species exhibited a typical angiosperm structure and gene composition, which could be classified into four categories: expression-related genes, photosynthesis-related genes, other genes, and genes of unknown function (Table 2). Gene duplication was observed primarily in the IR regions, resulting in two copies of rRNA genes (*rrn16S*, *rrn23S*, *rrn4.5S*, *rrn5S*), several tRNAs (*trnA* and *trnI*), and protein-coding genes (*ndhB*, *rpl2*, *rpl23*, *rps15*, *rps19*, *rps3*, and *rps7*). Several genes were found to contain introns, including single-intron genes (e.g., *ndhA*, *rps16*, *atpF*) and two-intron genes (e.g., *ycf3*).

### 2.2. Comparative Analysis and Nucleotide Diversity of Chloroplast Genomes

**Figure 2:**
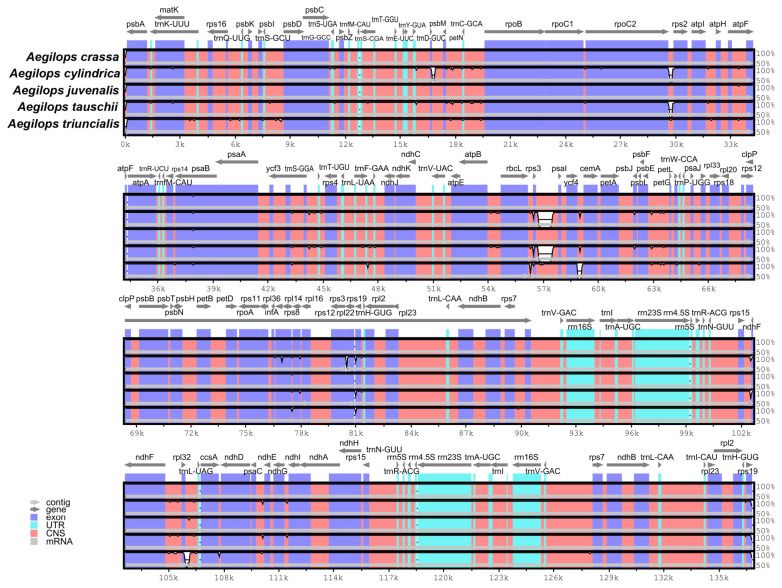
Comparative mVISTA analysis of chloroplast genomes of five *Aegilops* species. The horizontal axis shows positions within the chloroplast genome, while the vertical axis represents sequence identity percentages ranging from 50% to 100%.

The comparative mVISTA analysis revealed high sequence similarity among the five *Aegilops* chloroplast genomes, with most regions showing strong conservation (Figure 2). Coding regions were more conserved than non-coding regions, whereas higher variability was observed in intergenic spacers, particularly in the LSC and SSC regions. In contrast, the IR regions exhibited the highest sequence conservation.

**Figure 3:**
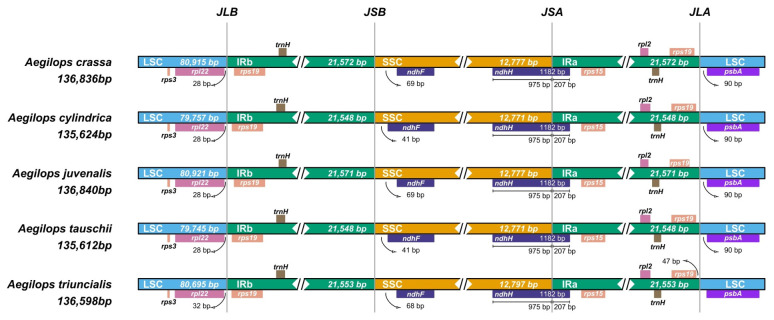
Inverted repeat (IR) boundary variation among five *Aegilops* chloroplast genomes. JLB, JSB, JSA, and JLA denote the LSC/IRb, SSC/IRb, SSC/IRa, and LSC/IRa junctions, respectively. Genes are represented by colored boxes, and the numbers shown above or below each gene indicate the distance between the gene ends and the corresponding junction boundaries.

Comparison of the IR/SC junctions in the five *Aegilops* chloroplast genomes showed a highly conserved structure (Figure 3). The LSC/IRb (JLB) junction was consistently located between the *rpl22* and *rps19* genes. At the same time, *rps19* was fully duplicated in the IR regions in all species. At the IRb/SSC (JSB) boundary, the *ndhF* gene extended into the SSC by 69 bp in *Ae. crassa* and *Ae. juvenalis*, 41 bp in *Ae. cylindrica* and *Ae. tauschii*, and 68 bp in *A. triuncialis*. The *ndhH* gene crossed the SSC/IRa (JSA) junction, while *rps15* was located within IRa in all species. The IRa/LSC (JLA) boundary was positioned between the *rps19* and *psbA* genes. Overall, small differences indicate slight IR expansion and contraction among species without major structural rearrangements.

**Figure 4:**
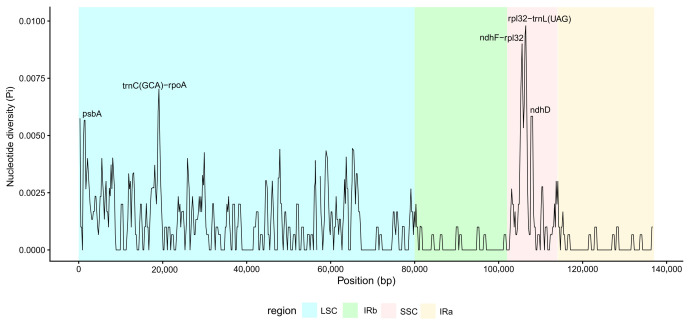
Sliding window analysis of nucleotide diversity across the chloroplast genomes of five *Aegilops* species. Window length: 600 base pairs; step size: 200 base pairs. The large single-copy (LSC), small single-copy (SSC), and inverted repeat (IR) regions are shown in different colors. The horizontal axis represents genomic regions, and the vertical axis indicates nucleotide diversity values.

Comparative analysis of nucleotide diversity (*Pi*) among the chloroplast genomes of five *Aegilops* species revealed generally low levels of sequence divergence across the genome (Figure 4). Sliding window analysis (window length = 600 bp, step size = 200 bp) showed that *Pi* values ranged from 0 to 0.00933, with an average nucleotide diversity of 0.00102. The highest nucleotide diversity was observed in the *rpl32-trnL(UAG)* intergenic region (*Pi* = 0.00933), followed by: *ndhF-rpl32* (*Pi* = 0.00800) and *trnC(GCA)-rpoA* (*Pi* = 0.00700) intergenic regions, *psbA* (*Pi* = 0.00567) and *ndhD* (*Pi* = 0.00567) genic regions. Regions with *Pi* values greater than 0.005 were considered relatively highly polymorphic and selected as candidate hotspot regions (Figure 4). These polymorphic regions were mainly located in the LSC and SSC regions, whereas the IR regions (IRa and IRb) exhibited markedly lower nucleotide diversity.

### 2.3. Simple Sequence Repeats (SSRs)

A total of 850 SSRs were identified across the chloroplast genomes of the five *Aegilops* species, with the number of SSRs in individual species ranging from 169 to 171 (Table S1). Among the different SSR types, mononucleotide repeats were the most abundant, accounting for 72.4% of all SSRs. Within this category, A/T repeats predominated (581 or 68.3%), whereas C/G repeats were much less frequent (35 or 4.1%).

**Figure 5:**
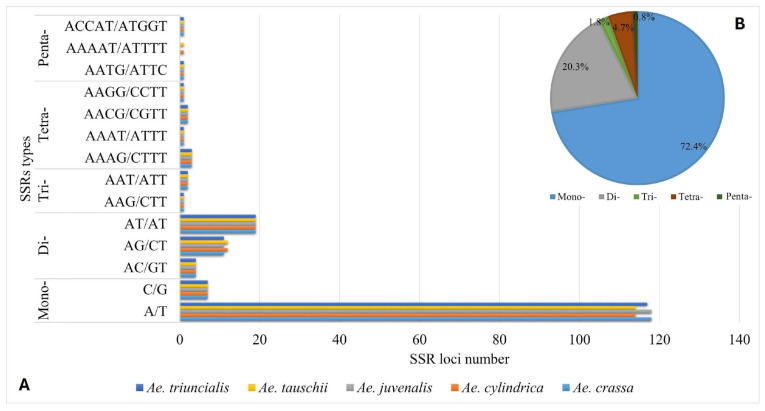
Distribution and composition of simple sequence repeats (SSRs) in the chloroplast genomes of five *Aegilops* species. (**A**) Distribution of SSR loci by repeat type and motif; (**B**) Proportion of different SSR types.

Dinucleotide repeats represented the second most common type, comprising 20.3% of the total SSRs. Among these, the AT/AT motif was the most frequent (95, 11.2%), followed by AG/CT (57, 6.7%) and AC/GT (20, 2.3%). Trinucleotide repeats were relatively rare, accounting for only 1.8% of all SSRs, including motifs such as AAG/CTT and AAT/ATT. Similarly, tetranucleotide repeats were present at low frequency (4.7%), with motifs such as AAAG/CTTT, AAAT/ATTT, AACG/CGTT, AAGG/CCTT, and AATG/ATTC identified across all species. Pentanucleotide repeats were the least abundant, representing only 0.8% of the total SSRs, and included motifs such as AAAAT/ATTTT and ACCAT/ATGGT (Figure 5).

### 2.4. Codon Usage and Ka/Ks Analysis

**Figure 6:**
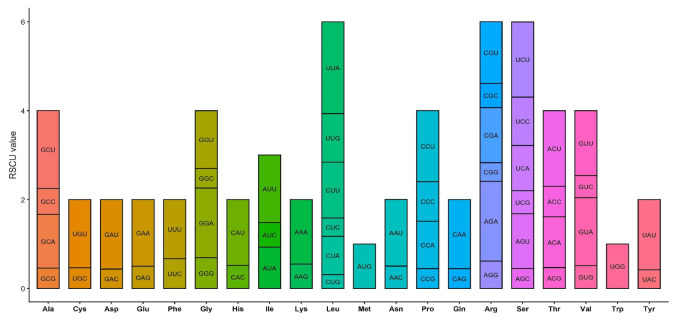
Codon usage bias in *Aegilops* based on relative synonymous codon usage (RSCU) values. The x-axis shows amino acid types, and the y-axis represents Relative Synonymous Codon Usage (RSCU) values.

The RSCU analysis identified a total of 61 sense codons across all examined *Aegilops* species, excluding stop codons, and revealed a pronounced and highly conserved codon usage bias among *Ae. crassa*, *Ae. cylindrica*, *Ae. juvenalis*, *Ae. tauschii*, and *Ae. triuncialis* (Table S2). Most amino acids exhibited unequal usage of synonymous codons, with several codons being overrepresented (RSCU > 1) and others underrepresented (RSCU < 1) (Figure 6). For fourfold degenerate amino acids such as Ala, Gly, Pro, and Thr, specific codons (e.g., GCU/GCC for Ala and GGU/GGC for Gly) were preferentially used, whereas others (e.g., GCG and GGG) were less frequent. Among twofold degenerate amino acids, a consistent preference for A/U-ending codons was observed, including GAU over GAC (Asp), GAA over GAG (Glu), UUU over UUC (Phe), and UAU over UAC (Tyr). A strong bias was also evident in sixfold degenerate amino acids, where Leu preferentially used UUA and UUG, while Arg favored CGU and CGC over AGA and AGG. Overall, the codon usage patterns were highly similar across all species, with a general preference for A/U-ending codons and an underrepresentation of G/C-ending codons, suggesting conserved nucleotide composition and similar evolutionary or translational selection pressures that shape codon bias in the genus *Aegilops*.

**Figure 7:**
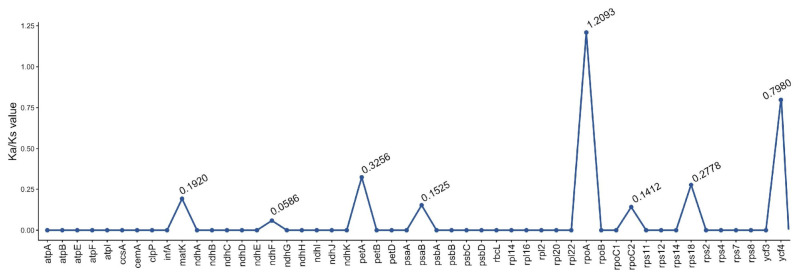
Variation in Ka/Ks ratios among 50 chloroplast protein-coding genes in *Aegilops* (CDS > 300 bp). The x-axis represents chloroplast protein-coding genes, while the y-axis shows the Ka/Ks ratio values.

A total of 50 CDS were used for the Ka/Ks analysis after removing genes shorter than 300 bp. The results showed that Ka/Ks values vary among the genes (Figure 7; Table S3). The majority of genes showed very low Ka/Ks values close to zero, suggesting that they are highly conserved. However, a few genes showed higher values. The highest Ka/Ks ratio was observed in *rpoA* (Ka/Ks = 1.2093), suggesting faster evolution. The gene *ycf4* also showed a relatively high value (0.7980). Moderate Ka/Ks values were observed in genes such as *petA* (0.3256), *rps18* (0.2778), and *matK* (0.1920), whereas *psaB* (0.1525) and *ndhF* (0.0586) showed slightly elevated but still relatively low values.

### 2.5. Phylogenetic Relationships

**Figure 8:**
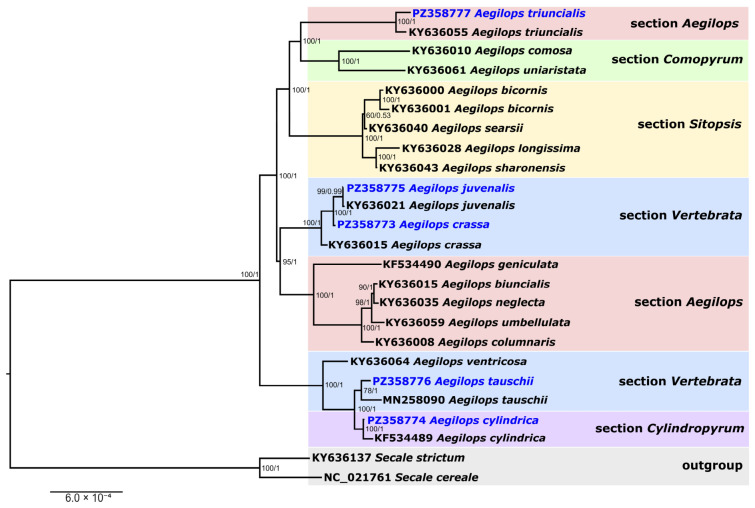
Consensus Maximum likelihood (ML) and Bayesian inference (BI) phylogenetic tree based on the complete chloroplast genome sequences of *Aegilops* species and outgroup taxa. The five *Aegilops* species sequenced in this study are highlighted in blue. Numbers on the branches indicate ML/BI values.

For the phylogenetic analysis of *Aegilops* species, Maximum Likelihood (ML) and Bayesian Inference (BI) methods were used to reconstruct phylogenetic trees. In addition to the five chloroplast genomes sequenced in this study, 18 previously published chloroplast genomes of *Aegilops* species and two *Secale* species were included in the analysis. The ML and BI trees showed largely congruent topologies and were therefore summarized as a consensus phylogenetic tree (Figure 8).

The resulting tree separated representatives of *Aegilops* into well-supported sectional clades, whereas *Secale strictum* and *S. cereale* formed a distinct outgroup lineage. The *Ae. triuncialis* accession sequenced in this study clustered with the reference sequence of *Ae. triuncialis* (KY636055) from GenBank within sect. *Aegilops*. Similarly, the *Ae. juvenalis* accession grouped with the reference *Ae. juvenalis* sequence (KY636021), and *Ae. crassa* clustered with *Ae. crassa* (KY636015), both within the sect. *Vertebrata*. The *Ae. tauschii* accession formed a highly supported clade with the reference *Ae. tauschii* sequence (MN258090), while *Ae. cylindrica* clustered with *Ae. cylindrica* (KF534489).

Most internal branches showed high bootstrap support and posterior probabilities, indicating strong support for the inferred relationships among the taxa analyzed. Overall, the tree topology was consistent with the sectional classification of the genus *Aegilops*, separating taxa belonging to the sects. *Aegilops*, *Comopyrum*, *Sitopsis*, *Vertebrata*, and *Cylindropyrum*. The close clustering of the chloroplast genomes generated in this study with their corresponding reference sequences confirms the accuracy of species identification and supports the utility of complete chloroplast genome sequences for resolving phylogenetic relationships within *Aegilops*.

## 3. Discussion

In this study, the complete chloroplast genomes of five *Aegilops* species were sequenced, assembled, and analyzed, including *Ae. crassa*, *Ae. cylindrica*, *Ae. juvenalis*, *Ae. tauschii*, and *Ae. triuncialis*. All five chloroplast genomes exhibited the typical quadripartite structure, comprising the LSC, SSC, and two IR regions. The genome sizes were highly conserved, ranging from 135,612 bp in *Ae. tauschii* to 136,840 bp in *Ae. juvenalis*, and all species had an identical GC content of 38% (Table 1). These findings are consistent with previous studies of Poaceae representatives, in which chloroplast genomes have also been reported to be highly conserved in both structure and gene content [31,52]. However, minor differences were observed in gene annotation among the studied species. The total number of annotated genes was generally similar, comprising 82 or 83 protein-coding genes, 37 tRNA genes, and 8 rRNA genes (Table 2). The difference in the number of protein-coding genes was attributable to variation in the duplication status of the *rps3* gene, which was not duplicated in the chloroplast genomes of *Ae. cylindrica* and *Ae. tauschii*. Similarly, the loss of the duplicated *rps3* copy has been reported in chloroplast genome sequences of 17 *Ae. tauschii* accessions [31].

Comparative analyses based on mVISTA (Figure 2) and IRscope (Figure 3) revealed no major structural rearrangements among the five *Aegilops* chloroplast genomes. The mVISTA results showed that the IR regions were more conserved than the LSC and SSC regions, which is a common feature of angiosperm chloroplast genomes [53,54]. Analysis of IR expansion and contraction further indicated a high degree of consistency among the *Aegilops* species studied. In particular, the distribution and positions of genes at the junctions of the LSC, SSC, and IR regions were highly similar across species. These findings are also consistent with previous studies reporting conserved chloroplast genome organization within closely related Poaceae taxa [55,56,57].

Highly variable regions are important sources of DNA barcoding markers for phylogenetic analyses and species identification. Chloroplast genome sequences play an important role in identifying potential DNA barcode loci because they provide variable regions suitable for phylogeny and distinguishing closely related taxa. In this study, five comparatively highly variable regions were identified, including three intergenic regions (*rpl32–trnL(UAG)*, *ndhF–rpl32*, and *trnC(GCA)–rpoA*) and two genic regions (*psbA* and *ndhD*) (Figure 4). These regions may serve as potential DNA barcodes for phylogenetic analysis and species identification within *Aegilops*. Among them, the *rpl32–trnL(UAG)* intergenic spacer showed the highest nucleotide diversity (*Pi* = 0.00933). This region has also been reported as suitable for phylogenetic studies in other *Aegilops* species [31,34]. The repeated identification of *rpl32–trnL* as a variable region suggests that this locus may be particularly useful for DNA barcoding and phylogenetic studies in *Aegilops*.

The SSRs are important molecular markers in plant population genetic studies and can provide valuable information for the conservation genetics of rare plant species [58,59]. SSRs identified from chloroplast genome sequences are widely used in population diversity analyses of various plant taxa [60,61]. Therefore, the identification of potential DNA markers, such as chloroplast SSRs, may provide useful tools for further studies in population genetics and conservation. In the present study, a total of 850 SSRs were identified across the five *Aegilops* chloroplast genomes, with the number of SSRs in individual species ranging from 169 to 171 (Table S1). Mononucleotide repeats were the most abundant type, particularly A/T repeats (Figure 5). Similar patterns have been reported in many chloroplast genome studies, in which A/T-rich mononucleotide repeats are usually dominant [62,63,64]. The chloroplast SSRs identified in this study could therefore be informative markers for future population genetic studies of *Aegilops* species.

Codon usage analysis showed a conserved pattern among all five species, with a general preference for A/U-ending codons (Figure 6). This is consistent with the AT-rich composition of chloroplast genomes and suggests that similar evolutionary pressures may shape codon usage in these species [65,66]. The similarity of codon usage among the five species also supports the conserved nature of chloroplast genomes in *Aegilops*.

The Ka/Ks analysis was performed using 50 CDS after excluding genes shorter than 300 bp. Most genes had Ka/Ks values below 1, indicating they are primarily under purifying selection. This means that most protein-coding genes in chloroplast genomes are functionally conserved and evolve slowly [66]. Similar patterns were reported in the *Triticum-Aegilops* complex, where relatively weak selection pressure on chloroplast coding genes was identified [33]. However, some genes of the five studied chloroplast genomes showed higher Ka/Ks values. The highest value was observed in *rpoA* (1.2093), followed by *ycf4* (0.7980), suggesting possible relaxed selection or faster evolution in these genes (Figure 7). Moderate values were observed in *petA*, *rps18*, and *matK*, while *psaB* and *ndhF* showed slightly elevated but still low values. These genes may deserve further attention in future studies with larger sample sizes.

Nucleotide sequences of complete chloroplast genomes are also valuable resources for plant phylogenomic studies [67]. Numerous studies have used chloroplast genome sequences to assess phylogenetic relationships [46,52], support species identification [68,69], and serve as super-barcodes to resolve relationships among plant taxa [70]. In the present study, complete chloroplast genomes were used to infer the phylogenetic relationships among *Aegilops* species. Phylogenetic trees were reconstructed using both ML and BI methods (Figure 7).

The topology of the obtained phylogenetic tree (Figure 8) is generally consistent with previous molecular studies of *Aegilops*. Sliai and Amer [71], using 5.8S rDNA and ITS2 sequences, showed that *Aegilops* species tend to cluster according to major species groups or sections, although relationships within some groups may vary depending on marker type and taxon sampling. Similarly, Bordbar et al. [21], using SSR, ITS, and chloroplast *trnL-F* sequences, reported close relationships among D-genome-bearing *Aegilops* species and highlighted a particularly close association among *Ae. crassa*, *Ae. juvenalis*, and *Ae. vavilovii*. The close placement of *Ae. crassa* and *Ae. juvenalis* in the present chloroplast genome tree is therefore consistent with earlier evidence from both nuclear and plastid markers. Bordbar et al. [21] also emphasized that D-genome-bearing *Aegilops* species show complex evolutionary patterns, including shared chloroplast haplotypes and evidence of chloroplast capture.

The newly sequenced *Ae. tauschii* chloroplast genome grouped with the reference *Ae. tauschii* sequence with strong support (Figure 8), confirming the accuracy of species identification and supporting its placement within the *Aegilops* phylogenetic framework. In addition, *Ae. cylindrica* clustered together with *Ae. tauschii*, indicating a close maternal relationship between these species [21,34]. This result is consistent with a previous study [21], which reported that *Ae. cylindrica* originated through hybridization between *Ae. markgrafii* (C genome) and *Ae. tauschii* (D genome), and that *Ae. tauschii* and *Ae. cylindrica* chloroplast haplotypes showed high homology, indicating *Ae. tauschii* as the maternal progenitor of *Ae. cylindrica*. Therefore, the clustering of *Ae. cylindrica* with *Ae. tauschii* in the chloroplast genome tree likely reflects their shared maternal lineage.

The chloroplast genome resources generated for five *Aegilops* species in this study provide useful DNA markers for phylogenetic and population genetic studies of the genus. In particular, the identified highly polymorphic regions and chloroplast SSR loci may serve as valuable molecular tools for species discrimination, phylogenetic analyses, and population-level investigations in *Aegilops*. Given the importance of *Aegilops* species as genetic resources for wheat improvement, these data may also support future germplasm characterization and breeding efforts. Furthermore, the chloroplast genomes generated from accessions collected in Kazakhstan and Uzbekistan expand the available genomic resources for Central Asian *Aegilops* germplasm.

Despite the increasing number of *Aegilops* chloroplast genome sequences available in NCBI GenBank, comprehensive comparative and phylogenetic studies based on complete chloroplast genomes remain limited. To date, phylogenetic relationships within *Aegilops* using complete chloroplast genome sequences have been investigated in only a few studies [31,34]. Moreover, many *Aegilops* chloroplast genome assemblies currently available in GenBank are incomplete, with lengths of approximately 114 kb. This limited the number of taxa that could be included in the comparative analyses performed in the present study. Another limitation is that chloroplast genomes represent only the maternally inherited component of the genome and therefore mainly reflect maternal evolutionary history. Consequently, chloroplast genome-based phylogenies should be interpreted together with nuclear genomic evidence, particularly in a genus such as *Aegilops*, where allopolyploidy, hybridization, and chloroplast capture have played important roles in species diversification [18,21]. Future phylogenomic studies integrating both nuclear and chloroplast genome data will help to further resolve evolutionary relationships and diversification patterns within *Aegilops*.

## 4. Materials and Methods

### 4.1. Plant Materials

Plant material from four *Aegilops* species (*Ae. triuncialis*, *Ae. cylindrica*, *Ae. crassa*, and *Ae. tauschii*) was collected in the Almaty and Turkestan regions of Kazakhstan, while *Ae. juvenalis* was collected in Uzbekistan (Table 3). In total, five *Aegilops* species were sequenced and analyzed in this study. Freshly collected leaves were dried in silica gel prior to DNA extraction. Genomic DNA was extracted from the dried *Aegilops* leaf material using the cetyltrimethylammonium bromide (CTAB) method [72].

### 4.2. Sequencing, Assembly, and Annotation

DNA samples that successfully passed quality control (QC) assessment were used for subsequent library construction. Paired-end libraries were prepared using the TruSeq Nano DNA Kit (Illumina Inc., San Diego, CA, USA). Chloroplast genome sequencing of five *Aegilops* species was performed on the Illumina NovaSeq 6000 platform (Illumina Inc., San Diego, CA, USA) at Macrogen (Seoul, Republic of Korea). The quality of the raw sequencing reads was evaluated using FastQC v0.11.7 (https://www.bioinformatics.babraham.ac.uk/projects/fastqc/, accessed on 10 April 2026). Adapter sequences and low-quality bases were removed using Trimmomatic v0.38 [73]. After quality filtering and adapter trimming, 36,718,702 reads were produced with a total of 5.5 Gbp for *Ae. triuncialis*, 38,397,536 reads were produced with a total of 5.7 Gbp for *Ae. cylindrica*, 36,224,364 reads were produced with a total of 5.4 Gbp for *Ae. crassa*, 36,816,438 reads were produced with a total of 5.5 Gbp for *Ae. tauschii*, and 37,049,802 reads were produced with a total of 5.5 Gbp for *Ae. juvenalis* were retained. The filtered reads showed high quality, with Q20 values of 99.6% for all samples and Q30 values ranging from 96.6% to 96.9%. De novo assembly of chloroplast genomes was performed using NOVOPlasty v4.3.3 [74], producing circular chloroplast genomes of 135,612–136,840 bp. Genome coverage was 100% for all assemblies, with average mapping depths ranging from 130.82× to 1078.71×. Genome annotation was performed using the GeSeq platform [75], followed by manual curation by comparison with a reference (*Ae. tauschii*, MN258081.1) retrieved from the National Center for Biotechnology Information (NCBI) GenBank database. Circular gene maps of the annotated chloroplast genomes were generated using the CHLOROPLOT online tool [76]. The assembled chloroplast genomes of the studied *Aegilops* species have been deposited in GenBank under accession numbers PZ358773–PZ358777.

### 4.3. Chloroplast Genome Comparative and Nucleotide Diversity Analysis

Comparative analysis of the chloroplast genomes of the studied *Aegilops* species was conducted using mVISTA [77] in Shuffle-LAGAN mode [78]. The contraction and expansion patterns of the inverted repeat (IR) regions at junction boundaries were examined and visualized using the IRscope online tool [79].

Nucleotide variability (*Pi*) was estimated using aligned chloroplast genome sequences in DnaSP v6 [80] with a sliding-window approach. The analysis employed a window size of 600 bp and a step length of 200 bp.

### 4.4. Simple Sequence Repeats Identification, Codon Usage, and Ka/Ks Analyses

Simple sequence repeats (SSRs, or microsatellites) within the chloroplast genomes of five *Aegilops* species (*Ae. triuncialis*, *Ae. cylindrica*, *Ae. crassa*, *Ae. tauschii*, and *Ae. juvenalis*) were identified using the MISA online tool [81], applying threshold settings of eight repeat units for mononucleotides, four for di- and trinucleotides, and three for tetra-, penta-, and hexanucleotide motifs.

For codon usage analysis, protein-coding genes were extracted from the chloroplast genomes of the five *Aegilops* species. A set of 50 shared coding sequences (CDS) was retained after removing overlapping genes and excluding sequences shorter than 300 bp, in accordance with established criteria [82]. Only non-redundant CDS with a valid ATG start codon and lengths exceeding 300 bp were used for subsequent analyses. Codon usage bias indices, including relative synonymous codon usage (RSCU) values [83], were calculated using CodonW v1.4.2 [84].

Pairwise nonsynonymous (Ka) and synonymous (Ks) substitution rates among the five studied *Aegilops* species were calculated using KaKs Calculator v2.0. The Ka/Ks ratios for the selected protein-coding genes were estimated with the Yang-Nielsen (YN) method implemented in the software [85].

### 4.5. Phylogenetic Analysis

Phylogenetic relationships were reconstructed using complete chloroplast genome sequences from 25 representatives of Poaceae. Among these, five *Aegilops* chloroplast genomes were generated in the present study, whereas 18 *Aegilops* genomes and two outgroup taxa were retrieved from NCBI GenBank (Table S4). Complete chloroplast genome sequences were aligned in Geneious Prime 2026.0.2 (https://www.geneious.com) using the default parameters. Phylogenetic analyses were conducted using two approaches: Maximum Likelihood (ML) and Bayesian Inference (BI). The ML analysis was performed in IQ-TREE 3 [86] under the best-fit substitution model K3Pu + F + I + R4, selected according to the Bayesian information criterion (BIC), with branch support assessed using 100,000 bootstrap replicates. BI was performed in MrBayes 3.2 [87] under the GTR + I + G nucleotide substitution model (lset nst = 6 rates = invgamma). For the BI analysis, posterior probabilities were estimated from two independent Markov Chain Monte Carlo (MCMC) runs of 10 million generations, sampling every 1000 generations. The first 25% of sampled trees were discarded as burn-in before summarizing the consensus tree. The resulting phylogenetic trees were visualized using FigTree 1.4.4 (https://github.com/rambaut/figtree/releases/tag/v1.4.4, accessed on 20 April 2026). Sectional and subgeneric classifications followed Schneider et al. [6].

## 5. Conclusions

In the present study, the complete chloroplast genomes of five *Aegilops* species (*Ae. crassa*, *Ae. cylindrica*, *Ae. juvenalis*, *Ae. tauschii,* and *Ae. triuncialis*) were sequenced, assembled, annotated, and comparatively analyzed. All chloroplast genomes exhibited the typical quadripartite structure and were highly conserved in genome size, GC content, gene content, and overall organization. Minor differences were primarily associated with the *rps3* gene’s duplication status, which was single-copy in *Ae. cylindrica* and *Ae. tauschii*. Nucleotide diversity analysis identified five comparatively variable regions, including *rpl32-trnL(UAG)*, *ndhF-rpl32*, *trnC(GCA)-rpoA*, *psbA*, and *ndhD*. A total of 850 chloroplast SSRs were detected, with A/T mononucleotide repeats being the most abundant. These variable regions and SSR loci may serve as useful molecular markers for future phylogenetic, DNA barcoding, and population genetic studies. Phylogenetic analyses based on complete chloroplast genomes showed that the ML and BI tree topologies were generally consistent with the sectional classification of *Aegilops*, supporting the usefulness of chloroplast genome data for species identification and phylogenomic studies within the genus. The newly generated plastome data in this study also expand the genomic resources currently available for *Aegilops*.

## Figures and Tables

**Table 1:** General characteristics of the chloroplast genomes among five *Aegilops* species.

Species Name	*Ae. crassa*	*Ae. cylindrica*	*Ae. juvenalis*	*Ae. tauschii*	*Ae. triuncialis*
Genome size	136,836	135,624	136,840	135,612	136,598
LSC region size	80,915	79,757	80,921	79,745	80,695
IRa region size	21,572	21,548	21,571	21,548	21,553
IRb region size	21,572	21,548	21,571	21,548	21,553
SSC region size	12,777	12,771	12,777	12,771	12,797
GC content, %	38	38	38	38	38
Total genes	128	127	128	127	128
CDS genes (duplicated)	83 (7)	82 (6)	83 (7)	82 (6)	83 (7)
tRNA genes (duplicated)	37 (8)	37 (8)	37 (8)	37 (8)	37 (8)
rRNA genes (duplicated)	8 (4)	8 (4)	8 (4)	8 (4)	8 (4)

**Table 2:** Functional classification and list of genes in the chloroplast genomes of five *Aegilops* species.

Category for Function	Function Groups for Genes	Genes
Expression	RNA polymerase	*rpoA*, *rpoB*, *rpoC1*, *rpoC2*
	Ribosomal proteins (LSU)	*rpl14*, *rpl16*, rpl2 * (×2), *rpl20*, *rpl22*, *rpl23* (×2), *rpl32*, *rpl33*, *rpl36*
	Ribosomal proteins (SSU)	*rps2*, *rps3* (×2) ψ, *rps4*, *rps7* (×2), *rps8*, *rps11*, rps12 *, *rps14*, *rps15* (×2), rps16 *, *rps18*, *rps19* (×2)
	Transfer RNAs	trnA-UGC * (×2), *trnC-GCA*, *trnD-GUC*, *trnE-UUC*, *trnF-GAA*, *trnG-GCC*, *trnH-GUG* (×2), trnI * (×2), *trnI-CAU*, trnK-UUU *, *trnL-CAA* (×2), trnL-UAA *, *trnL-UAG*, *trnfM-CAU* (×2), *trnN-GUU* (×2), *trnP-UGG*, *trnQ-UUG*, *trnR-ACG* (×2), *trnR-UCU*, trnS-CGA *, *trnS-GCU*, *trnS-GGA*, *trnS-UGA*, *trnT-GGU*, *trnT-UGU*, *trnV-GAC* (×2), trnV-UAC *, *trnW-CCA*, *trnY-GUA*
	Ribosomal RNAs	*rrn16S* (×2), *rrn23S* (×2), *rrn4.5S* (×2), *rrn5S* (×2)
Photosynthesis	Photosystem I	*psaA*, *psaB*, *psaC*, *psaI*, *psaJ*
	Photosystem II	*psbA*, *psbB*, *psbC*, *psbD*, *psbE*, *psbF*, *psbH*, *psbI*, *psbJ*, *psbK*, *psbL*, *psbM*, *psbN*, *psbT*, *psbZ*
	ATP-dependent protease subunits P gene	*clpP*
	ATP synthase	*atpA*, *atpB*, *atpE*, atpF *, *atpH*, *atpI*
	Cytochrome b/f complex	*petA*, *petB*, *petD*, *petG*, *petL*, *petN*
	NADH dehydrogenase	ndhA *, ndhB *(×2), *ndhC*, *ndhD*, *ndhE*, *ndhF*, *ndhG*, *ndhH*, *ndhI*, *ndhJ*, *ndhK*
	Large subunit of Rubisco	*rbcL*
Other genes	C-type cytochrome synthesis gene	*ccsA*
	Envelope membrane protein	*cemA*
	Maturase	*matK*
	Translation initiation factor	*infA*
Unknown function	Conserved open reading frames	ycf3 **, *ycf4*

Notes: *—genes containing introns; **—genes containing two introns; (×2)—duplicated genes; ψ rps3 is duplicated only in *Ae. crassa*, *Ae. juvenalis*, and *Ae. triuncialis*, whereas a single copy is present in *Ae. cylindrica* and *Ae. tauschii*.

**Table 3:** Locations of sampled *Aegilops* species.

Species	Latitude	Longitude	Elevation	Year of Collection	Collection Sites
*Aegilops triuncialis*	41.480411	69.154039	431	2025	Turkestan region, Saryagash district
*Aegilops cylindrica*	43.536378	77.477263	563	2025	Almaty region, Enbekshikazakh district
*Aegilops crassa*	42.11838	68.11657	400	2015	Turkestan region, Bairkum district
*Aegilops tauschii*	43.288200	76.259033	768	2009	Almaty region, Zhambyl district
*Aegilops juvenalis*	38.134731	66.455522	1133	2009	Uzbekistan, Dekhkanabad district

## Data Availability

The data presented in this study are openly available in the National Center for Biotechnology Information (NCBI) GenBank database at https://www.ncbi.nlm.nih.gov, reference numbers PZ358773–PZ358777.

## Supplementary Materials

The following supporting information can be downloaded at: https://www.mdpi.com/article/10.3390/ijms27135680/s1.
