# Supplementary figures and images for: Chloroplast Genome Characterization, Comparative Analysis, and Phylogenetic Insights into Five Aegilops Species
Source: Int J Mol Sci. 2026 Jun 24;27(13):5680. doi: 10.3390/ijms27135680 (PMC13362065; doi:10.3390/ijms27135680)

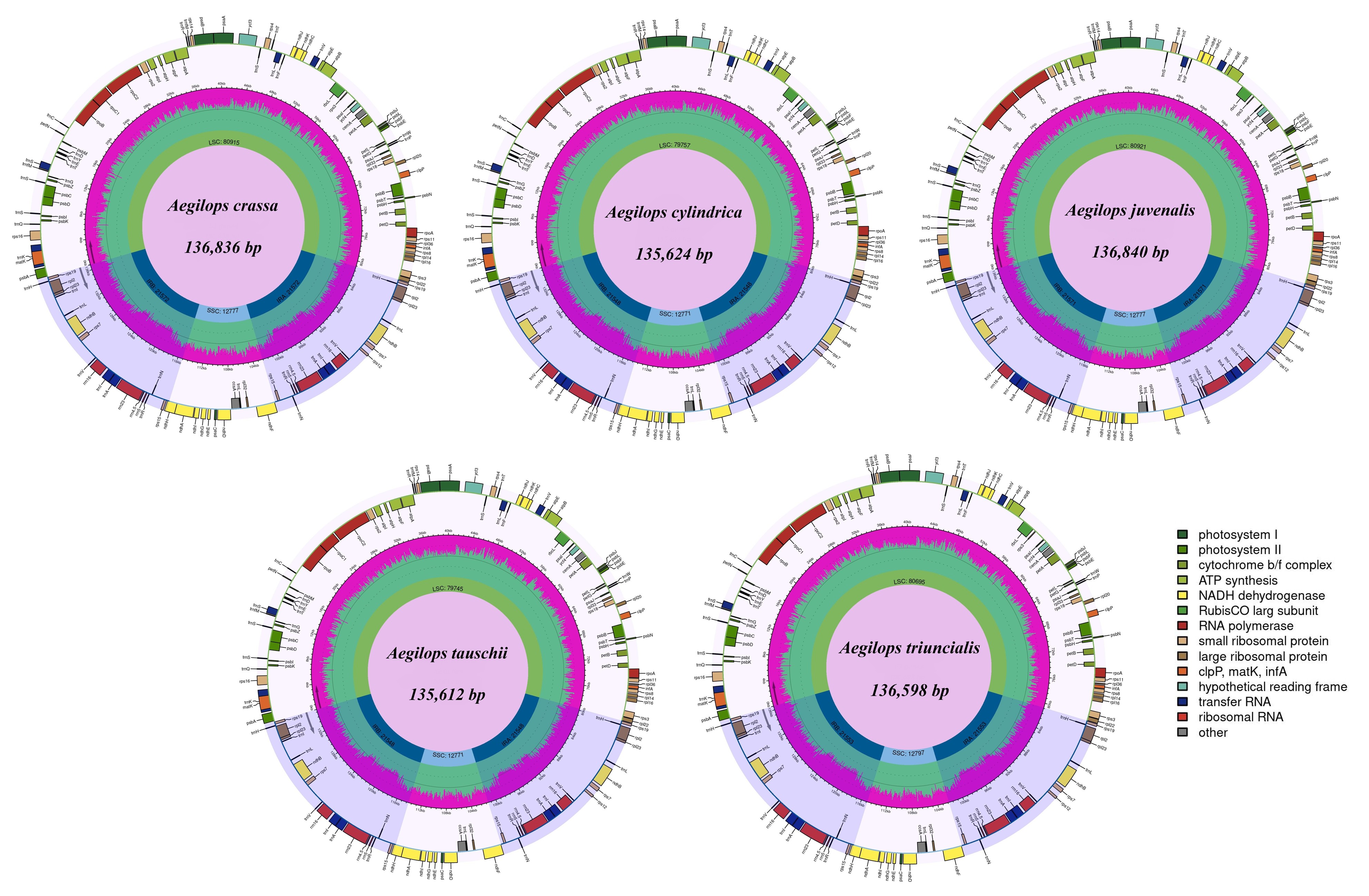

Supplement: Supplementary file 1 [file ijms-27-05680-s001.zip › Figure S1.jpg]
